# Vitamin D Responsive Elements within the *HLA-DRB1* Promoter Region in Sardinian Multiple Sclerosis Associated Alleles

**DOI:** 10.1371/journal.pone.0041678

**Published:** 2012-07-25

**Authors:** Eleonora Cocco, Alessandra Meloni, Maria Rita Murru, Daniela Corongiu, Stefania Tranquilli, Elisabetta Fadda, Raffaele Murru, Lucia Schirru, Maria Antonietta Secci, Gianna Costa, Isadora Asunis, Stefania Cuccu, Giuseppe Fenu, Lorena Lorefice, Nicola Carboni, Gioia Mura, Maria Cristina Rosatelli, Maria Giovanna Marrosu

**Affiliations:** 1 Centro Sclerosi Multipla, Dipartimento di Sanità pubblica, Medicina clinica e molecolare, University of Cagliari, Cagliari, Italy; 2 Istituto di Ricerca Genetica e Biomedica, Consiglio Nazionale delle Ricerche (CNR), Cagliari, Italy; 3 Centro di Psichiatria e Psicosomatica Azienda Ospedaliera Universitaria, Cagliari, Italy; 4 Dipartimento di Sanità pubblica, Medicina clinica e molecolare, University of Cagliari, Cagliari, Italy; Roswell Park Cancer Institute, United States of America

## Abstract

Vitamin D response elements (VDREs) have been found in the promoter region of the MS-associated allele HLA-DRB1*15∶01, suggesting that with low vitamin D availability VDREs are incapable of inducing *15∶01 expression allowing in early life autoreactive T-cells to escape central thymic deletion. The Italian island of Sardinia exhibits a very high frequency of MS and high solar radiation exposure. We test the contribution of VDREs analysing the promoter region of the MS-associated DRB1 *04∶05, *03∶01, *13∶01 and *15∶01 and non-MS-associated *16∶01, *01, *11, *07∶01 alleles in a cohort of Sardinians (44 MS patients and 112 healthy subjects). Sequencing of the *DRB1* promoter region revealed a homozygous canonical VDRE in all *15∶01, *16∶01, *11 and in 45/73 *03∶01 and in heterozygous state in 28/73 *03∶01 and all *01 alleles. A new mutated homozygous VDRE was found in all *13∶03, *04∶05 and *07∶01 alleles. Functionality of mutated and canonical VDREs was assessed for its potential to modulate levels of *DRB1* gene expression using an *in vitro* transactivation assay after stimulation with active vitamin D metabolite. Vitamin D failed to increase promoter activity of the *04∶05 and *03∶01 alleles carrying the new mutated VDRE, while the *16∶01 and *03∶01 alleles carrying the canonical VDRE sequence showed significantly increased transcriptional activity. The ability of *VDR* to bind the mutant VDRE in the *DRB1* promoter was evaluated by EMSA. Efficient binding of *VDR* to the VDRE sequence found in the *16∶01 and in the *15∶01 allele reduced electrophoretic mobility when either an anti-*VDR* or an anti-*RXR* monoclonal antibody was added. Conversely, the Sardinian mutated VDRE sample showed very low affinity for the RXR/VDR heterodimer. These data seem to exclude a role of VDREs in the promoter region of the *DRB1* gene in susceptibility to MS carried by DRB1* alleles in Sardinian patients.

## Introduction

Multiple sclerosis (MS) is a chronic inflammatory and degenerative disease of the central nervous system. Although the etiology of MS remains unknown [1], it is commonly believed that genetic susceptibility combined with exposure to environmental factors are required for its development [2]. Data emerging from geographical, biological and immunological studies suggest that among the non-genetic risk factors, vitamin D may be one of the key determinants for the development of MS [3], [4]. Epidemiological studies have demonstrated that MS incidence follows a latitude gradient in both hemispheres, being more common in northern regions in Europe and North America and in the southern part of the Australia [5]. In addition to the geographical distribution of MS prevalence, other data as influence on MS risk according to season of birth [6]–[9] and differential risk in migrants [3] suggest that alongside ethnic differences in population structure, sun-mediated photosynthesis of vitamin D plays a role in promoting the distribution MS gradient.

The population of Sardinia, Italy’s second largest island is genetically characterized by a low degree of large scale genetic heterogeneity, and by a distribution of alleles at multiple loci different from other Europeans [10]. The incidence and prevalence rates of MS on the island are among the highest in the world [11]. It has been suggested that this phenomenon may arise from the particular genetic structure of the population [12]. A complex multi-locus and multi-allelic disease association with the main genetic effects encoded by variation at the HLA class II loci *DRB1* and *DQB1* has been found in Sardinia. In particular, the association is carried by the DRB1*13∶03-DQB1*03∶01, DRB1*04∶05-DQB1*03∶01, DRB1*03∶01-DQB1*02∶01, DRB1*15∶01-DQB1*06∶02 and DRB1*04∶05-DQB1*03∶02 haplotypes [13]. Recently, the positive association with the DRB1*13∶03-DQB1*03∶01, DRB1*04∶05-DQB1*03∶01 and DRB1*03∶01-DQB1*02∶01 haplotypes has been confirmed along with a negative (i.e., protective) association with the DRB1*1601-DQB1*0502 haplotype [14]. The main predisposing haplotype in Northern European populations, (*DR2*) DRB1*15∶01-DQB1*06∶02 [15], although very rare in the Sardinian population, is still significantly positively associated with MS [13], [14]. The frequencies of the MS-associated haplotype DRB1*03∶01-DQA1*05∶01-DQB1*02∶01 and the MS protective haplotype DRB1*16∶01-DQA1*01∶02-DQB1*05∶02 are the highest so far reported in any ethnic group (haplotype frequency 21.9% and 19.1%, respectively) [16]. By contrast, the DRB1*15∶01-DQA1*01∶02-DQB1*06∶02 haplotype, which has a high frequency in other ethnic groups, is rare in Sardinia (haplotype frequency 1.5%) [16]. Also the *HLA-DR4* haplotypes show a peculiar distribution in Sardinians. The *04∶01 allele, which is the most common *DR4* subtype in other Caucasian populations, is absent in Sardinians. Only *DR4* haplotypes bearing the *04∶05, *04∶02 and *04∶03 alleles at the *DRB1* locus are common variants in this population [16]. Furthermore, Sardinians, in contrast to other Caucasian populations, have virtually only one DR4-DQB1*03∶01 haplotype in which the DQ-α chain is encoded by the DQA1*05∶01 gene (i.e., the DRB1*04∶05-DQA1*05∶01-DQB1*03∶01 haplotype) [16].

Despite the multiallelic risk profile identified in Sardinian MS patients [13], [14], genetic variation within the HLA region can only partially explain the high propensity to the disease in Sardinians, both in terms of content and relative contributions of the disease associated alleles and haplotypes.

Sardinia is the second largest island in the Mediterranean Sea with a surface of 24,090 km^2^; it is located between 38° 51′ 52″ and 41° 15′ 42″ latitude north and 8° 8′ and 9° 50′ east longitude. Solar radiation levels are high with about 300 days of sunlight a year: a median of 4–6 hours in November and the winter months, 10 hours in spring and 11 hours in September, October and the summer months. Because ultraviolet radiations (UVR) are the major source of vitamin D in humans, and the demonstration that UVR suppress experimental autoimmune encephalomyelitis (EAE) independently of vitamin D production [17], the high prevalence of MS on the island seems to be a paradox.

The human body has a self-regulatory system by which it produces adequate amounts of vitamin D. Vitamin D can either be synthesized in the skin or obtained from dietary intake [18]. Recently, the active metabolite of vitamin D the 1,25-dihydroxyvitamin D3 (1,25-(OH)_2_-D_3_) has been shown to be involved in cell proliferation and differentiation, immune suppression, as well as gene expression in different cells [19], [20].

1,25-(OH)_2_-D_3_ acts as ligand for the vitamin D receptor (*VDR*), a member of the nuclear receptor superfamily of transcriptional regulators of vitamin D-dependent gene expression [21]. Binding of 1,25-(OH)_2_-D_3_ induces conformational changes in the *VDR* that promote heterodimerization of *VDR* with the retinoid X receptor (*RXR*), followed by translocation of the complex into the nucleus. The VDR/RXR complex binds to vitamin D responsive elements (VDREs) in the promoter region of 1,25-(OH)_2_-D_3_ target genes [21] which, in turn, results in regulatory function of 1,25-(OH)_2_-D_3_. Upon binding to VDREs, the heterodimer RXR/VDR/1,25-(OH)_2_-D_3_ activates or suppresses gene transcription, thus inducing or repressing protein synthesis [20]. A VDRE generally consists of two direct imperfect repeats of six nucleotides separated by a three-nucleotide spacer. The *VDR* occupies the 3′ half-site, while the *RXR* occupies the 5′ half-site. Sequence variations in the 3′ half-element, the 5′ half-element and the spacer and in sequences flanking the VDREs appear to be important in determining receptor-binding efficiency [22]. The presence and location of VDREs have been described only in a small proportion of genes transcriptionally regulated by 1,25-(OH)_2_-D_3_ [23]–[26]. A VDRE, identified in the proximal promoter region immediately 5′ to the transcriptional start site of *HLA-DRB1*, has been found to be highly conserved in the MS-associated *15∶01 allele. No VDREs have been identified in the non-MS-associated *04, *07, and *09 haplotypes carried by populations of Northern-European descent [27]. Transfection and flow cytometric assays have shown that the VDRE present in the *HLA-DRB1* promoter influences gene expression and imparts 1,25-(OH)_2_-D_3_ sensitivity to the *15 allele. This supports the interesting hypothesis that vitamin D deficiency (by insufficient dietary intake and/or sunlight exposure) in the uterus or during early childhood may affect expression of *HLA-DRB1* in the thymus, allowing autoreactive T cells to escape thymic deletion [27].

Because the high UVR in Sardinia, the high prevalence of MS and the peculiar DRB1* alleles MS- association, Sardinia appears as an ideal setting to investigate the VDREs in the promoter region of the *DRB1* and test the above mentioned hypothesis [27]. Thus, we investigated the presence and functionality of VDREs in the *DRB1* promoter region of Sardinian MS-associated, protective and neutral HLA-DRB1* haplotypes [13], [14]. Sequencing of the promoter region of predisposing and non-predisposing *DRB1* alleles, showed functionally conserved VDREs in two of three *03∶01 alleles, the most frequent MS-associated allele in the Sardinian population, and in the *16∶01 allele, the most frequent protective allele in the same population [13], [14]. A non functional mutated VDRE was found in the promoter region of the MS-associated *04∶05 and *13 alleles and in one of three *03∶01 alleles, as well as in all neutral *0701 allele. On the basis of these data, we propose that in Sardinian MS population VDREs in the promoter region of the *DRB1* gene do not influence susceptibility to the disease.

## Results

### *In silico* Identification of Putative Vitamin D Response Elements

The presence of VDREs in the promoter region of the MS-positively (*13∶03-*03∶01, *04∶05-*03∶01, *03∶01-*02∶01 and *15∶01-*06∶02) and negatively (*1601) and non-associated alleles (*11, *01 and *07) [13], [14] has been studied. Like *15∶01, the *16∶01 allele is a serological split of *HLA-DR2*, and represents the second most frequent allele in the Sardinian healthy population (frequency 19,1%) [16].

Sequences were scanned *in silico* for VDREs using JASPAR_CORE version 3.0 database with a profile score threshold of 80% [28]. Analysis with these settings localised only one putative VDRE to the proximal promoter region of *HLA-DRB1*.

### Sequencing of the HLA-DRB1 Promoter in MS Patients and Controls

The presence and conservation of the putative VDRE element was examined in 156 individuals of Sardinian descent (44 MS patients and 112 healthy controls) within four of the five major groups of *HLA-DR* haplotypes, i.e. DR1 (DRB1*01), DR51 (DRB1*15, *16), DR52 (DRB1*03, *11, *13) and DR53 (DRB1*04, *07).

The *DRB1* promoter was sequenced in 9 *01, 3 *15∶01, 15 *16∶01, 73 *03∶01, 16 *11, 2 *13, 30 *04∶05, and 8 *07∶01 homozygous individuals, both MS affected and unaffected (Table 1).

**Table 1:** Sequencing of the *HLA-DRB1* promoter region in 156 subjects of Sardinian descent (112 healthy individuals and 44 multiple sclerosis affected individuals) carrying *HLA-DRB1*-*DQA1-DQB1* haplotypes.

		HLA DRB1 haplotypes	156 homozygousSubjects
DRB1*	DQB1*		
01∶01	05∶01	HLA-DR1	9 (5.8%)
01∶02	05∶01		
01∶03	05∶01		
03∶01	02∶01	HLA-DR52	73 (46.8%)
04∶05	02∶01	HLADR53	30 (19.2%)
04∶05	03∶02		
04∶05	03∶01		
07∶01	02∶01	HLA-DR53	8 (5.1%)
07∶01	03∶03		
11∶01	03∶01	HLA-DR52	16 (10.3%)
11∶04	03∶01		
15∶01	06∶02	HLA-DR51	3 (1.9%)
16∶01	05∶02		15 (9.6%)
13		HLA-DR52	2 (1.3%)

The putative canonical VDRE GGGTGGAGGGGTTCA was present in all *15∶01, *16∶01 and *11 bearing haplotypes, with no variants disrupting the 15 base pair VDRE consensus sequence.

In 45 of the 73 *03∶01 homozygous individuals, the putative canonical VDRE was found in the promoter region of both chromosomes, while the 28 remaining individuals had heterozygous sequences in the promoter region, showing not only the canonical sequence, as above, but also a new sequence GAGTAGAGGGAGGTCA characterized by three mismatches in the consensus sequence for the RXR/VDR heterodimer and by a 4 nucleotide spacer between the two repeats.

This new sequence segregated in all *13, *04∶05 and *07∶01 carriers, while it was present in heterozygosis with the canonical sequence in all *01 carriers (Table 2).

**Table 2:** Distribution of VDRE sequences in the promoter region of *HLA-DRB1* alleles.

	GGGTGGAGGGGTTCA Canonical VDRE			GAGTAGAGGGAGGTCANew 16 nt sequence					
DRB1*	Homozygous			Homozygous			Heterozygous		
	MS	Healthy	Tot	MS	Healthy	Tot	MS	Healthy	Tot
01∶0	0	0	0	0	0	0	1	8	9
03∶01	15	30	45	0	0	0	9	19	28
04∶05	0	0	0	9	21	30	0	0	0
07∶01	0	0	0	4	4	8	0	0	0
11∶0	3	13	16	0	0	0	0	0	0
15∶01	2	1	3	0	0	0	0	0	0
16∶01	1	14	15	0	0	0	0	0	0
13∶0	0	0	0	0	2	2	0	0	0

**Figure 1:**
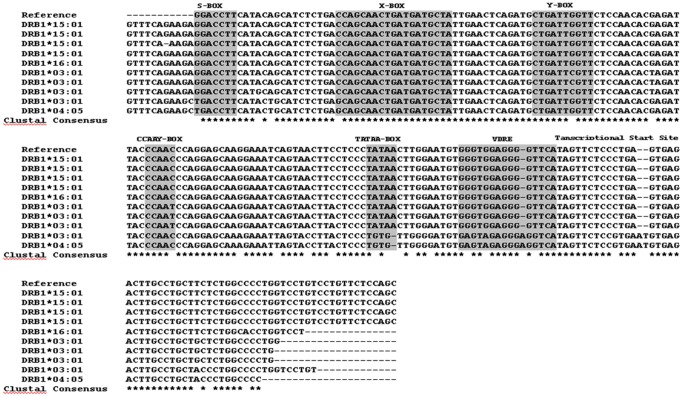
Sequences of the proximal promoter region of DRB1*15∶01, DRB1*16∶01, DRB1*03∶01 and DRB1*04∶05 **alleles.** The important regulatory elements S, X, Y, CCAAY, TATA-BOX and VDRE are highlighted. Note the presence of two different sequences in the VDRE region, the canonical one and a 16 nucleotide variant. Stars (*) in the last row show homology and empty spaces show nucleotide substitution in one or more samples at that particular site and dashes(−) represent gaps inserted to maximize the homology. The sequences have been aligned with reference sequence from The IMGT/HLA Database.

Figure 1 shows the proximal promoter region of *15∶01, *16∶01, *03∶01 and *04∶05 alleles. The important regulatory elements S, X, Y, CCAAY, TATA-BOX and VDRE are highlighted. Note the presence of two different sequences in the VDRE region: the canonical sequence in *15∶01, *16∶01 and three of four *03∶01 alleles, and the 16 nucleotide variant in *04∶05 and one of four *03∶01 alleles that is moreover linked with a disrupted TATA box (TGTG).

### Transactivating *in vitro* Activity of *DRB1* Promoters

The *DRB1* promoter region of five individuals, three homozygous for the *03∶01-*02∶01 haplotype, one homozygous for the *04∶05-*03∶01 haplotype and one homozygous for the *16∶01-*05∶02 haplotype, was cloned using the TOPO-TA Cloning® kit (Invitrogen Corporation Carlsbad, California 92008) and then sequenced.

Three individuals (two carrying the *03∶01-*02∶01 haplotype and one carrying the *16∶01-*05∶02 haplotype) were homozygous for the putative canonical GGGTGGAGGGGTTCA VDRE sequence (sample 1910B). These findings overlap with the results obtained for the MS-associated *15∶01 allele in the Canadian population [27]. Differently, the *04∶05-*03∶01 haplotype displayed the new non canonical VDRE sequence GAGTAGAGGGAGGTCA (sample 585C), described above. In the third *03∶01-*02∶01 sample both sequences were found in the compound heterozygous state.

The highly polymorphic VDRE identified in the *DRB1* promoter of Sardinian MS patients was then investigated for its potential to modulate levels of *DRB1* gene expression. An *in vitro* transactivation assay was developed after stimulation with active vitamin D metabolite.

Reporter gene constructs from sample 1910B (putative canonical sequence: GGGTGGAGGGGTTCA) and sample 585C, (mutated sequence: GAGTAGAGGGAGGTCA) were designed so that −181 to +53 of the *HLA-DRB1* gene sequence was placed upstream of a pGL3 luciferase reporter gene.

The constructs were transfected in cell lines expressing vitamin D receptor. A renilla luciferase reporter construct was co-transfected to normalise luciferase activity. In order to avoid the masking effect of endogenous hormones on vitamin D response, cell cultures were carried out in a medium containing hormone-free fetal bovine serum.

The promoter region of the *16∶01 and *03∶01 alleles carrying the canonical VDRE sequence showed significantly increased transcriptional activity. Also in *15∶01 carriers of the Canadian population [27], this VDRE sequence is an active regulatory element of promoter activity.

Vitamin D treatment shows just a minor positive effect on the promoter activity of the *04∶05 and *03∶01 alleles carrying the new non canonical VDRE sequence.

**Figure 2:**
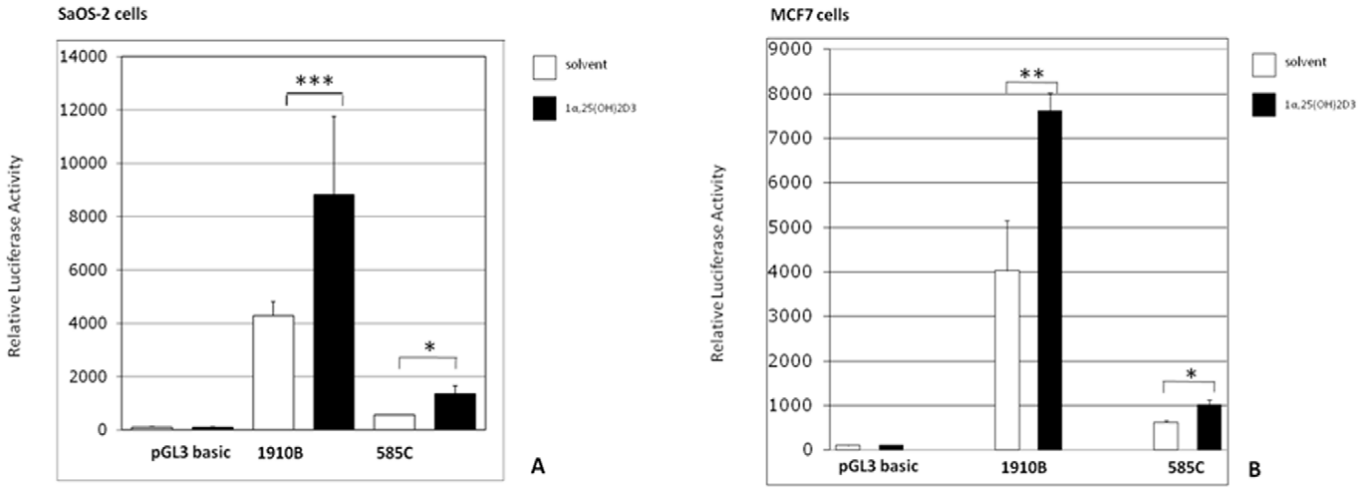
Functionality of the putative VDREs (samples 1910B *03∶01 and 585C *04∶05) identified within the promoter region of the *HLA-DRB1* gene. Reporter gene assay was performed with extracts from SaOS-2 cells (A) and MCF-7 cells (B) that were transiently transfected with *luciferase* reporter constructs each containing the promoter region of the *HLA-DRB1* gene. Cells were treated for 16 h with either solvent or 100 nM 1α, 25(OH) 2D3. Columns represent means of at least three experiments performed in triplicate. Two-tailed t test were performed to determine the significance of the increased response to calcitriol treatment (*p<0,05; **p<0,001; ***p<0,0001).

Data are shown in Figure 2.

### *In vitro* Binding of *VDR* to the *DRB1* Vitamin D Responsive Elements

**Figure 3:**
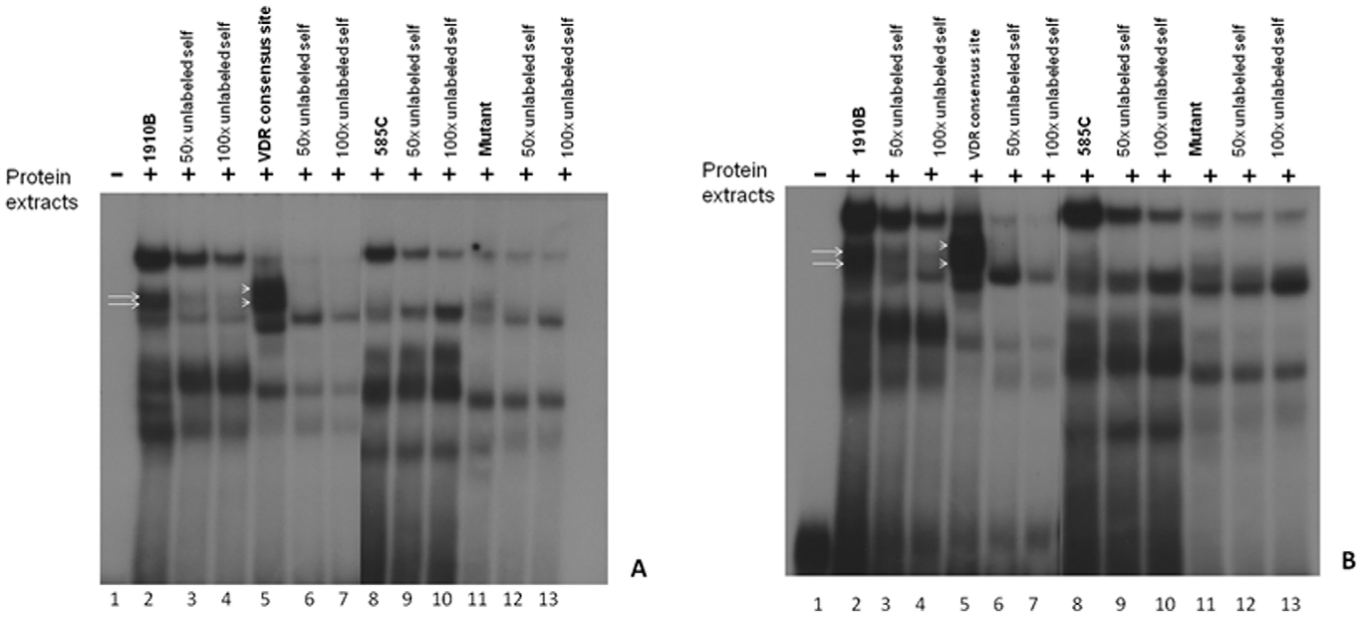
In vitro binding of VDR protein to the putative binding site of the *HLA-DRB1* promoter in MCF7 and SaOS-2 cells. A strong interaction between VDR and the radiolabelled probes corresponding to both the VDRE consensus sequence (line 5) and the 1910B *03∶01 sequence (line 2), was detected by electrophoretic mobility shift assay in both MCF7 (A) and SaOS-2 (B) nuclear cell extracts. White arrows indicate RXR-VDR (top) and VDR–VDR (bottom) complexes. Both complexes were specifically competed by a 100-fold molar excess of each sequence-specific unlabelled probe (Lanes 3–4 and 6–7). Sample 585C *04∶05 and the negative control mutant (lanes 8 and 11, respectively) did not bind to VDR.

The ability of *VDR* to bind the mutant VDRE in the Sardinian *HLA-DRB1* promoter, was evaluated by *in vitro* electrophoretic mobility shift assay (EMSA) (Figure 3).

The two previously described VDRE sequence motifs GGGTGGAGGGGTTCA, sample 1910B (*03∶01 homozygous allele), and GAGTAGAGGGAGGTCA, sample 585C (*04∶05 homozygous allele), were included in the experiment.

A canonical consensus sequence for *VDR* was used as positive control, while a totally disrupted *RXR* binding sequence, including a 4-nucleotide spacer, was used as negative control.

**Figure 4:**
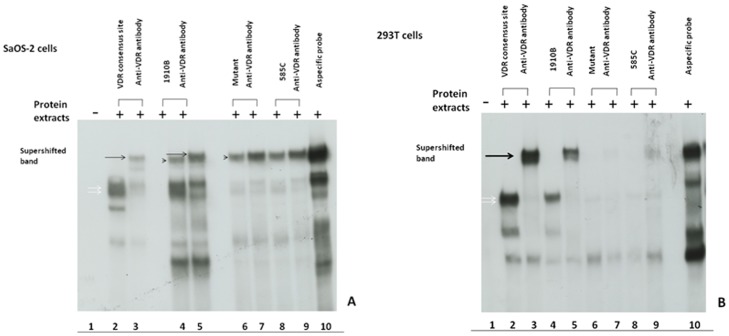
Supershift assay in SaOS-2 cells and 293T transfected cells. In SaOS-2 cells, antibody directed against VDR protein resulted in retarded electrophoretic mobility of RXR-VDR and VDR-VDR complexes in lanes 3 and 5, corresponding to VDRE canonical consensus site (positive control) and the 1910B *03∶01 sample. White arrows indicate RXR-VDR (top) and VDR-VDR (bottom) complexes. The thin arrow indicates the supershifted band, while the short arrow indicates the aspecific band. In 293T cells transfected with VDR and RXR constructs, the supershifted band is observed in line 3 for VDRE canonical consensus site (positive control) and line 5 for 1910B *03∶01 sample.

The results of this experiment clearly demonstrated efficient binding of RXR-*VDR* heterodimer to the VDRE sequence GGGTGGAGGGGTTCA (sample 1910B) as well as to the canonical consensus sequence (positive control). In the supershift assay performed in Saos-2 cells and 293T transfected cells both complexes showed reduced electrophoretic mobility when either an anti-*VDR* or an anti-*RXR* monoclonal antibody was added (Figure 4). Additionally, both complexes were specifically competed with 100-fold molar excess of unlabelled VDRE probe (Figure 3).

Conversely, the probe corresponding to the Sardinian mutant VDRE sample 585C showed very low affinity for the RXR/VDR heterodimer, probably because of the 3 mismatches in the *RXR* binding site. The weak and poorly detectable signal showed in the figure is most likely due to binding of a *VDR* monomer.

## Discussion

It is widely accepted that MS risk arises from a combination of genetic and environmental factors. Among genes involved in MS susceptibility, the *HLA-DRB1* locus has been the most consistent determinant of risk worldwide [29]. However, while in the majority of geographically prone areas the disease is associated with *15∶01 [15], [29], in Sardinia the *DRB1* association is carried by different haplotypes. The DRB1*13∶03-DQB1*03∶01, DRB1*04∶05-DQB1*03∶01 and DRB1*03∶01-DQB1*02∶01 haplotypes are positively associated whereas the DRB1*16∶01-DQB1*0∶502 haplotype is negatively associated and other haplotypes, such as *01, *11 and *07 are not associated (neutral) [14]. Among the environmental factors, vitamin D has been proposed to have a key role in the risk of developing MS [30]. UVR, the major source of vitamin D, decreases with increasing latitude, leading to the long-lasting speculation that low sunlight exposure is related to MS gradient [31]. Moreover, a recent study reported that individuals with the highest residential and occupational solar exposure have the lowest rate of MS incidence [32]. In addition, role of vitamin D in MS is indicated by a number of in vivo studies, demonstrating that 1,25(OH)_2_D_3_ suppress disease induction and progression in the EAE model [33]–[35]. However, it is unclear if UVR, vitamin D or both are necessary for the putative decrease in susceptibility to MS, because clinical signs of EAE were suppressed by UVR treatment independently of vitamin D production, likely through different mechanisms than 1,25(OH)_2_D_3_ [17].

Sardinia, where high MS prevalence [11] and high UVR coexist, is a notable exception to the epidemiological data correlating low sunlight exposure with high MS prevalence and *vice-versa* [36]. A gene-environmental link involving poor UVR, vitamin D and the *15∶01 predisposing allele has been hypothesized to explain the high prevalence of MS in population of North European descent. Indeed, Ramagopalan et al. found a functional VDRE in the promoter region of the *15∶01 allele, the expression of which was influenced by 1,25-(OH)_2_-D_3_, suggesting that low levels of vitamin D (by insufficient dietary intake and/or sunlight exposure) in the womb or early in life can impair expression of *15∶01 in the thymus, allowing autoreactive T cells to escape thymic deletion [27].

Because the concomitance of the peculiar MS-DRB1* allelic association [13], [14], the high prevalence of the disease and the high UVR in the island, we tested the above mentioned hypothesis exploring the presence and functionality of VDREs in the promoter region of the *DRB1 alleles conferring or not an increased MS risk in the Sardinian population. Sequencing of associated and non-associated alleles revealed canonical VDREs in two of three *03∶01 susceptibility alleles and in all protective *16∶01 and *11 non-associated alleles, whereas a novel mutated VDRE was found in the promoter region of the *13 and *04∶05 predisposing alleles, other than in one of three *03∶01 alleles and the *01 and *07∶01 non-associated alleles. Transactivation assays after stimulation with active vitamin D metabolite showed significantly increased transcriptional activity of *DRB1* promoters containing the GGGTGGAGGGGTTCA putative canonical VDRE sequence, which thus acts in the same way as the identical responsive sequence found in *15∶01 carriers of the Canadian population [27].

On the other hand, vitamin D treatment failed to increase promoter activity of *04∶05 and *03∶01 alleles carrying the new mutated VDRE sequence. This sequence consistently differs from the consensus sequence RGKTSANNNRGKTSA for the RXR-VDR heterodimer, even bringing a deeply disrupted TATA box.

These results were confirmed by EMSA showing very low affinity of the mutant VDRE in the Sardinian *HLA-DRB1* promoter for the RXR/VDR heterodimer. Conversely, *VDR* bound, as expected, to the putative canonical VDRE.

Autoimmune diseases are characterized by a breakdown in the mechanisms of tolerance to self-antigens. Central self-tolerance during T-cell differentiation in the thymus involves deletion (negative selection) of self-reactive T-cells [37]. The currently favoured model for cellular interactions leading to positive and negative selection of thymocytes is the quantitative/avidity model, where cell fate is determined by cumulative thymocyte interactions that are in part governed by avidity [38]. According to this model, weak interactions between the T-cell receptor (TCR) and self peptide/MHC complexes lead to positive selection while stronger avidity interactions lead to negative selection. Hence, the fate of the thymocyte is determined by interactions of T-cell receptor (TCR) with the self MHC/peptide complexes expressed on thymic stromal cells. Thymocytes expressing high-affinity/avidity TCRs for peptide/MHC complexes undergo apoptosis and are deleted centrally in the thymus, whereas those bearing TCRs those bind too weakly or not at all survive negative selection. Therefore, among the signalling pathways mediating positive and negative selection, the structure and expression of self-MHC molecules play a crucial role [39]–[41].

Based on the presence of functional VDREs in the promoter region of *15∶01 allele, the hypothesis that in low condition of UVR and vitamin D availability the expression of the at risk allele can be impaired, allowing to a defective thymic T cell selection [27], make sense. However, findings obtained in Sardinian population seem to contradict the above mentioned hypothesis: a mutated, not functional VDRE was found in Sardinian predisposing *04∶05, *13∶03 and in two over three *03∶01 alleles as in all protective *16∶01 and *11 neutral alleles, while canonical and functional VDRE was present in one over three predisposing *03∶01 allele as in *01 and *07∶01 neutral alleles. Thus, it is likely that in Sardinia expression of DRB1* at risk alleles is independent of VDREs and that vitamin D synthesis by UVR do not influence the propensity to MS observed in the island. Variability in vitamin D status and MS relapse risk has been reported in different populations: for instance, a serum level of 95nM is associated with relapse in Argentina [42], but with remission in Finland [43]. Threshold for vitamin D deficiency determining clinical and immunological effect may differ according to evolutionary selection in human populations for VDR gene and for VDREs present in the human genome [44]–[45].

In the pathogenesis of another autoimmune disease, type 1 diabetes (T1D), inherited variation of vitamin D and low levels of circulating vitamin D have been reported [46]. A functional VDRE has been found in the promoter region of the at risk *03∶01 allele in a population of patients from North India [47], allowing the authors to speculate that in condition of low vitamin D levels the expression of *03∶01 is insufficient to reach the threshold of thymic deletion, similarly to that hypothesized in MS [27]. Sardinia is also a high-risk area for T1D [48]. Interestingly, a consistent hierarchy for the HLA class II genetic determinants of T1D has been reported in Sardinian T1D patients: the risk of *04∶05-*03∶02 carriers is more than two-fold the risk of *03∶01 ones, whereas the *16∶01 and, more consistently, the *15∶01 alleles have a protective effect (RR = 0.8 and 0.04, respectively) [49]. There are no studies exploring the influence of VDREs on *DRB1* expression of at risk alleles in Sardinian T1D patients, but it is likely that the same mutated and not functional VDRE can be found in the at risk *04∶05* as in some *03∶01 alleles, while canonical and functional VDREs should be present in the protective *16∶01 and *15∶01 alleles. If this hypothesis is confirmed, it could be a further demonstration that in Sardinian population mechanisms driving the expression of DRB1* associated alleles in autoimmune diseases are independent from VDRE in the promoter region of the *DRB1* gene.

In conclusion, our data suggest that MS susceptibility in the Sardinian population is independent of VDREs in the promoter region of the associated DRB1* alleles. Presently, there are no data about levels of vitamin D in both healthy and MS Sardinian individuals. The apparent paradox of MS prevalence on the island, despite high UVR, remains unexplained. Other factors, as conformational characteristics of predisposing and non-predisposing molecules intervening in self-peptide binding, molecular mimicry of exogenous peptides from pathogens such as Mycobacterium avium paratuberculosis, very common in the island and recently involved in MS pathogenesis [50], could account for the role of specific DRB1* alleles in conferring risk to MS in Sardinia.

## Materials and Methods

### Subjects

Unrelated subjects (MS patients and healthy subjects) homozygous at the HLA DRB1 locus were randomly selected from the DNAs collection of the MS Center of Cagliari. In it are stored more than 3000 and 1500 DNAs coming respectively from MS patients and from demographically and ethnically matched healthy subjects (HS). All the individuals considered in the study were born in Sardinia and were Sardinians at least from three generations. Totally 156 subjects were included, of them 44 were MS patients, of them 37 were female and 7 were male; all but 1 have a Relapsing remitting/secondary progressive course, the mean age at onset was 29 SD +/−9.3 years; the age at the time of the study was 41 SD +/−12.3 years. MS patients were ascertained and followed up at the Multiple Sclerosis Centre of Cagliari (Sardinia) and the disease was diagnosed according to the McDonald criteria [51]. The 112 HS were 48 male and 64 female the mean actual age of HS was 42.5 SD +/−13.5 years. Moreover, they had no autoimmune diseases and any relatives affected with MS.

The study was conducted in accordance with the Helsinki Declaration and approved by University of Cagliari/ASL8 (Italy) ethics committee. All subjects gave informed written consent.

### *HLA-DRB1* Genotyping and Sequencing

The *HLA-DRB1* promoter region was sequenced in 156 subjects of Sardinian descent (113 healthy subjects and 43 MS patients) recruited by the Regional Center for Multiple Sclerosis in Cagliari (Southern Sardinia). The female to male ratio was 1.35 in healthy samples and 2.58 in MS patients. Among the 43 samples from MS affected individuals, 19 came from trios families, whereas 24 were independent cases. All subjects were homozygous for *HLA-DRB1* alleles. *HLA-DRB1* genotyping was performed as previously described [13], [14].

Two primers selected with Primer3 (v. 0.4.0) were used to amplify a 374 bp fragment in the *HLA-DRB1* promoter region. The amplified products were sequenced to determine the VDRE variants in the Sardinian population.

An ABI 3130xl genetic analyser was used to process sequencing samples prepared with ABI’s BigDye® Terminator v3.1 sequencing chemistry (Applied Biosystems, Inc., Carlsbad, California).

The sequences were then analysed by Applied Biosystems Sequence Scanner Software v1.0.

Sequences were aligned using ClustalW2, and the presence of a VDRE was confirmed in-silico with the JASPAR_CORE version 3.0 database using default conditions [28].

Finally, all sequences were confirmed by dot spot hybridization with probes complementary to the TATA box and VDREs. All methods are available upon request.

### Plasmid Constructs

The promoter region (from nt−181 to +53) of two patients, one (1910B *03∶01 homozygous allele) containing the VDRE sequence GGGTGGAGGGGTTCA corresponding to the Canadian responsive sequence in the homozygous state and the other (585C *04∶05 homozygous allele) homozygous for the non canonical VDRE sequence GAGTAGAGGGAGGTCA, were amplified by PCR and cloned into XhoI/HindIII sites in the pGL3 basic vector upstream of the luciferase gene.

### Cell Culture, Transfection and Luciferase Reporter Gene Assay

MCF7 and SaOS-2 cells were seeded into 6-well plates (10 ^5^ cells/ml) and grown overnight in phenol red-free DMEM supplemented with 10% charcoal-treated fetal bovine serum. Liposome complexes were formed by incubating 1 µg of reporter control vector phRL-TK (PROMEGA) and 4 µg of pGL3 *DRB1* promoter vector with Lipofectamine 2000 reagent for 15′ at room temperature in a total volume of 100 µl [52]. After dilution with 900 µl of phenol red-free DMEM, the liposomes were added to each of the cell cultures. Phenol red-free DMEM supplemented with 30% charcoal-treated fetal bovine serum was added 4 h after transfection. At the same time, 100 nM 1,25(OH)2D3 (calcitriol) or solvent were also added. The cells were lysed 16 h after onset of stimulation using passive lysis buffer (PROMEGA). Luciferase assay was performed on cell lysates using the Dual-Luciferase Reporter®, Assay kit from Promega according to the manufacturer’s instructions. Luminescence was measured in a Microlumat LB96P luminometer (EG&G Berthold, Wildbad, Germany).

Luciferase activity was normalized with respect to renilla luminescence produced by reporter control vector phRL-TK. Induction factors were calculated as the ratio of luciferase activity of ligand-stimulated cells to that of solvent control. The procedure was the same for both cell lines.

The Saos-2 osteosarcoma and the MCF-7 mammary gland breast cells were obtained by The American Type Culture Collection (ATCC; number HTB-85 and CRL-2351 respectively).

### Gel Shift and Supershift Assay

The EMSA experiments were conducted on SaOS-2, MCF7 and 293T cell cultures. The 293T cells were transfected with the constructs pCMV6-XL5-VDR (ORIGENE Technologies) and pSG5-RXR (donated by professor P. Moi, University of Cagliari). After stimulation with vitamin D, the cells were collected and the nuclear proteins were extracted using the Nuclear extraction kit (Active Motif). Two micrograms of each nuclear protein extract was incubated with 1ng of the ^32^P-labeled VDRE consensus probe and with each of its radiolabelled variant. Incubation was performed in binding buffer (25 mM Hepes (K+) ph7,5, 100 mM KCl, 12,5 mM MgCl2, 1 mM DTT, 10 µM ZnSO4, 20% glicerol, 0,1% Nonidet P-40) for 20 min. at room temperature. In the supershift assay, 10 µl of anti-VDR and anti RXR antibody (Santa Cruz) were added to the mixture. In the competition assay, 100 ng of cold probe were added. Following incubation, the reactions were analysed as autoradiograms of 6% no denaturing polyacrylamide gels. The 293T kidney cells were obtained by ATCC (number CRL-11268).
